# In search of the mRNA modification landscape in plants

**DOI:** 10.1186/s12870-019-2033-2

**Published:** 2019-10-14

**Authors:** Jagna Chmielowska-Bąk, Magdalena Arasimowicz-Jelonek, Joanna Deckert

**Affiliations:** Department of Plant Ecophysiology, Institute of Experimental Biology, Faculty of Biology, Adam Mickiewicz University, ul. Uniwersytetu Poznańskiego 6, 61-614 Poznań, Poland

**Keywords:** RNA modifications, Epitranscriptomics, Methylation, Oxidation, Nitration, Modified ribonucleotides

## Abstract

**Background:**

Precise regulation of gene expression is indispensable for the proper functioning of organisms in both optimal and challenging conditions. The most commonly known regulative mechanisms include the modulation of transcription, translation and adjustment of the transcript, and protein half-life. New players have recently emerged in the arena of gene expression regulators – chemical modifications of mRNAs.

**Main text:**

The latest studies show that modified ribonucleotides affect transcript splicing, localization, secondary structures, interaction with other molecules and translation efficiency. Thus far, attention has been focused mostly on the most widespread mRNA modification – adenosine methylation at the N^6^ position (m^6^A). However, initial reports on the formation and possible functions of other modified ribonucleotides, such as cytosine methylated at the 5′ position (m^5^C), 8-hydroxyguanosine (8-OHG) and 8-nitroguanosine (8-NO_2_G), have started to appear in the literature. Additionally, some reports indicate that pseudouridine (Ψ) is present in mRNAs and might perform important regulatory functions in eukaryotic cells. The present review summarizes current knowledge regarding the above-mentioned modified ribonucleotides (m^6^A, m^5^C, 8-OHG, 8-NO_2_G) in transcripts across various plant species, including Arabidopsis, rice, sunflower, wheat, soybean and potato.

**Conclusions:**

Chemical modifications of ribonucleotides affect mRNA stability and translation efficiency. They thus constitute a newly discovered layer of gene expression regulation and have a profound effect on the development and functioning of various organisms, including plants.

## Background

The first information about RNA modification appeared in the mid-twentieth century and described the occurrence of pseudouridine (Ψ) in tRNA. It was initially called a “fifth nucleotide” [1, 2]. Shortly afterwards, ribonucleotide modifications were found in other RNA types, including messenger RNA. In the 1970s, the formation of 7-methylguanosine in the process of mRNA capping was first reported [3]. Simultaneously, methylation of adenosine in the N^6^ position was detected for the first time in mRNAs [4–6]. Further progress in the study of modified ribonucleotides was hindered for nearly 50 years due to a lack of advanced molecular biology techniques. Research has accelerated significantly in recent years with the development of new technologies, including next-generation sequencing (NGS) and bioinformatic tools. Today, the database of RNA modifications, MODOMICS, includes over 160 modified ribonucleotides [7]. Although the exact functions of many of them are still elusive, it is known that modified nucleotides can affect mRNA’s secondary structure, stability and interaction with other molecules, such as rRNA, tRNA and RNA binding proteins (RBP). The consequent downstream effects include the modulation of transcript processing, half-life, transport and translation efficiency, all of which have a profound effect on cellular functioning [8, 9]. Some crucial processes, such as cell differentiation, development, sexual determination, circadian rhythm, stress response and pathogenesis are regulated by mRNA chemical modifications [10, 11].

**Fig. 1:**
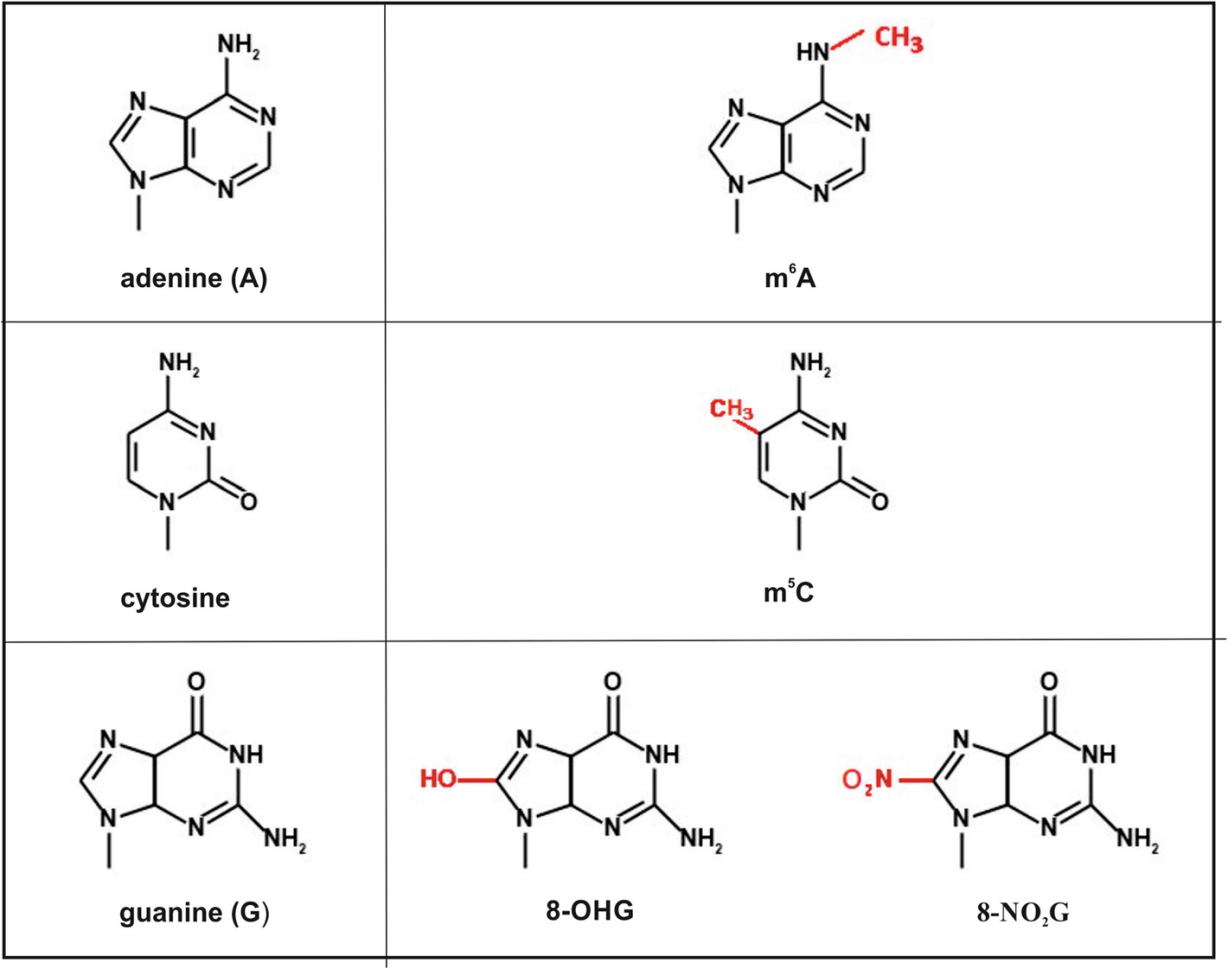
Examples of modified ribonucleotides detected in plants mRNA; m^6^A - N^6^-methyladenine; m^5^C - 5-methylcytosine; 8-OHG - 8-hydroxyguanine; 8-NO_2_-G - 8-nitroguanine

Although most research dedicated to the topic of epitranscriptomics has been carried out on animal and human models, significant progress in this field has also been made in plants. Modifications in various plant RNA types have been recently reviewed by Burgess, David and Searle [12]. The present article is focused exclusively on modified ribonucleotides in mRNA. It presents up-to-date information on adenine methylated at the N^6^ position (m^6^A), cytosine methylated at the 5′ position (m^5^C), 8-hydroxyguanosine (8-OHG) and 8-nitroguanosine (8-NO_2_G) (Fig. 1), highlighting their profound role in the regulation of gene expression, plant development and stress response .

### Messenger RNA methylation

Methylation of adenosine at the N^6^ position (m^6^A) is the most widely studied internal mRNA modification. It was first detected in the 1970s in oat, maize and wheat plants [13–15]. However, progress made in sequencing, bioinformatic and genetic engineering techniques has allowed for more detailed insight into the distribution and possible functions of m^6^A.

Recent reports show that adenosine methylation is particularly extensive – it is found in 70–77% of the Arabidopsis transcripts. Even higher methylation rates have been noted in particular organelles – this modification was found in 86–90% of transcripts in mitochondria and 98–100% in chloroplasts. Methylated adenosine is present mainly in the consensus RRm^6^ACH sequence (where R stands for A or G, and H stands for A, C or T), near the stop codon and in the 3′ UTR regions [16–20], although some reports show that it may also occur near the start codon [21]. The level of m^6^A is transient and depends on the activity of the enzymatic machinery, consisting of so-called “writers”, which catalyze the methylation process, and “erasers” mediating adenosine demethylation*.* Additionally, a third group of proteins, named “readers”, is important for m^6^A metabolism. These proteins bind to methylated adenosine and mediate downstream effects. Readers usually contain the YTH domain engaged in m^6^A recognition [17, 22]. The identified plant writer, eraser and reader proteins and the impact of their down- or up-regulation on plant phenotype is presented in Table 1.

**Table 1:** Proteins engaged in m^6^A metabolism in plants

Abbreviation	Full name	Plant Species	Functions	Cellular localization	Phenotype of the mutant with decrease expression
MTA	mRNA adenosine methylase	Arabidopsis (*Arabidopsis thaliana*)	form m^6^A methylation complex	nucleus	*• mta*, *mtb*, *fip37* and *vir* are embryo-lethal *•* mature *mta*, *mtb*, *fip37* and *vir* plants show alerted vascular development, gravitropic response, roots growth and flowering timing *• mta* mutants are characterized by small, bushy rosettes and alerted flower and trichome architecture *• hakai-1* and *hakai-2* mutants resembled wild type plants [23–27],
MTB					
FIP37	FKB12 Interacting Protein 37 kD				
VIR	VIRILIZER				
HAKAI					
ECT1–13	FKB12 Interacting Protein 37 kD	Arabidopsis (*Arabidopsis thaliana*)	confirmed or putative m^6^A binding proteins engaged in downstream responses including regulation of mRNA lifetime	Various YTH-domain proteins were detected in cytoplasm and nucleus or ubiquitously in cells, depending on the plant species and specific protein	*• ect2* mutant showed increased trichome branching *•* double ect2/ect3 mutants show increased trichome branching and delayed leave formation accompanied by changes in leaves shape [28–34]
MhYTP1–15	YTH-domain protein	Chinese crab apple (*Malus hupehensis*)			
OsYTH1–12	YTH-domain protein	Rice (*Oryza sativa*)			
CsYTH1–5	YTH-domain protein	Cucumber (*Cucumis sativus*)			
ALKB10B	Alpha-ketoglutarate-dependent dioxygenase 10B	Arabidopsis (*Arabidopsis thaliana*)	mediates m^6^A demethylation	not examined	*•* hampered growth and delayed flowering [21]

Precise regulation of the m^6^A metabolism is indispensable for proper plant development. Studies on Arabidopsis and rice indicate that m^6^A formation is associated with the development of organ-specific functions. For example in rice selectively methylated genes (SMG) in the leaves encode proteins engaged in photosynthesis, while SMGs in callus encode proteins involved in the regulation of transcription, nucleic acid binding and nitrogen metabolism [19]. Alterations in methylation/demethylation machinery lead to changes in the stability of the transcript involved in developmental processes and, consequently, to severe developmental defects, including embryo-lethal phenotype, disturbed development of vascular tissues, hampered inflorescence internodes and root growth, altered structure of the shoot apical meristem (SAM), leaf rosettes and trichome branching, changes in the timing of flower development and disturbances in flower architecture [21, 23–27].

The downstream effects of adenosine methylation in transcripts is most likely dependent on the type of binding reader protein [21, 25]. According to the “N^6^-methyladenosine-dependent RNA structural switches” model, adenosine methylation leads to the remodeling of mRNA and increases its binding capacity with specific reader proteins, usually members of the YTH family [35]. Studies on human and animal models show that, depending on the associated YTH proteins, m^6^A might affect mRNA metabolism in many distinct ways – by increasing its translocation from nucleus to cytoplasm, modulating stability, enhancing translation or affecting splicing [8]. Research in recent years have yielded an abundance of studies concerning proteins belonging to the YTH-domain family in plants, including Arabidopsis, rice, Chinese crab apple and cucumber (listed in Table 1). The most studied protein is probably the Evolutionary Conserved C-terminal Region 2 (ECT2) protein in Arabidopsis. It has been shown that ECT2 binds to m^6^A enriched regions and stabilized transcripts involved in trichome development such as *Transparent Testa Glabra1* (*TTG1*), *Irregular Trichome Branch1* (*ITB1*) and *Distorted Trichomes2* (*DIS2*). Loss of ECT2 function leads to alerted trichome development reflected in increased branching [28–30]. The variable expression of proteins belonging to YTH domain family in response to stress factors indicates that, in addition to their engagement in developmental processes, these proteins might also play a role in plants’ reaction to unfavorable conditions [31–33]. This assumption is further strengthened by the fact that introduction of apple *MhYTP1* and *MhYTP2*, belonging to YTH domain proteins, into *Arabidopsis* plants resulted in higher tolerance to salinity and drought [34]. Taken together, the described examples show that m^6^A is an extensive modification found in the majority of mRNAs, and that it affects plant development through modulation of the stability of specific transcripts. Yet it is quite likely that its role is more complex, as observed in the case of human and animal cells. For instance, the co-localization of elements of the adenosine methylation complex in splicing speckles indicates the putative engagement of m^6^A in pre-mRNA splicing [24]. However, thus far this function has not been confirmed experimentally in plants.

Although m^6^A is the most abundant methylation-dependent mRNA modification, other methylated ribonucleotides might also have a significant effect on the functioning of plant cells [18]. Indeed the process of cytosine methylation in the 5′ position (m^5^C) in mRNA has been reported in Arabidopsis (*Arabidopsis thaliana*), maize (*Zea mays*), rice (*Oryza sativa*), foxtail millet (*Sateriaitalica*) and barrel clover (*Medicago truncatula*), with the latter showing the highest level. Formation of this modification is dependent on the activity of tRNA specific methyltransferase 4 (TRM4) and is modulated by various external factors, such as drought, heat and treatment with phytohormones. A decreased level of m^5^C is associated with reduced root length, hampered cell proliferation and increased sensitivity to oxidative stress, indicating its role in the regulation of plant development and oxidative response [36, 37]. However, the exact mechanism of this phenomenon is thus far unknown. Intriguing reports on m^5^C and RNA: m^5^C methyltransferase (RCMTs) functions come from studies on human cells. The results of recent research carried out on leukaemia cells indicate that nascent RNA containing m^5^C may form complexes with specific RCMT and other associated proteins. The formed complex interacts with chromatin and affects its structure. Depending on the type of bound RCMT, these changes in chromatin structure induce sensitivity or resistance to azacitidne (5-AZA) – one of the drugs used for treatment of haematologic malignancies [38]. It is thus highly likely that complexes formed by m^5^C RNA and additional proteins modulate chromatin structure and in consequence its binding capacity and possibly transcriptional activity.

### Oxidative mRNA modifications

The formation of reactive oxygen species (ROS) likely accompanied the appearance of the first oxygen molecules 2.4–3.8 billion years ago. In excess, these highly reactive compounds lead to damage in biological molecules, including proteins, lipids and nucleic acids. However, ROS are also engaged in crucial cellular processes, such as signaling, differentiation, defense responses and programmed cell death. Therefore, maintaining adequate ROS levels is indispensable for the proper functioning of organisms [39]. Numerous modifications of nucleic acids might be formed as a result of ROS action [40]. The most common ones include 8-hydroxydeoguanosine (8-OHdG) in DNA and 8-hydroxyguanosine (8-OHG) in RNA. It was initially believed that their occurrence is a symptom of oxidative damage. However, recent reports suggest that their role might be much more complex.

A high frequency of 8-OHG in human mRNA is associated with the development of neurodegenerative and neurodevelopmental disorders, including Alzheimer’s and Parkinson’s disease, amyotrophic lateral sclerosis (ALS), dementia of Lewy bodies, schizophrenia, epilepsy and prion disease [11, 41]. Elevated levels of this mRNA oxidation marker were also noted in patients suffering from Down syndrome, lung emphysema, hereditary hemochromatosis and type II diabetes [41, 42]. Additionally, 8-OHG is suggested to be a marker of aging. A study carried out on over 1000 people showed a correlation between an increase in urinary 8-OHG levels and age. The level of the marker increased nearly twofold in the case of people aged 81–90 years when compared to those aged 11–20 years [43]. Similarly, yeast mutants characterized by premature aging showed significantly higher levels of 8-OHG in comparison to the wild type [44]. The association between 8-OHG formation and degenerative processes might indicate that RNA oxidation is a symptom of pathogenesis and damage. However, some studies point out that the occurrence of this modification precedes the development of actual pathogenic symptoms. It has been additionally shown that it is a selective process limited to defined transcripts and that it leads to hampered translation of specific proteins [45, 46]. Even more interestingly, in plants 8-OHG formation in transcripts was first observed during a normal process in the plant’s life cycle: the breaking of seed dormancy. In this case, the process was also limited to a defined transcript and resulted in a decreased level of encoded proteins. For instance, in the case of nondormant sunflower seeds, the 8-OHG rich transcripts were associated with metabolism, transport and stress response. In turn, in wheat seeds subjected to after-ripening processes, oxidation occurred mainly in transcripts encoding proteins engaged in the regulation of nutrient storage and α-amylase activity. The results suggest that oxidation of transcripts might be important for the regulation of seed germination. According to this hypothesis, the increased oxidation of mRNAs associated with the dormancy process (for example *α-amylase/trypsin* inhibitor and *starch synthase*) leads to a decreased level of encoded proteins and the breaking of seed dormancy [47, 48]. The exact role of RNA oxidation in plants’ stress response has not yet been elucidated. It has been shown that 8-OHG formation in mRNA and/or total RNA is induced by exposure to cadmium in soybean and nematode infection in Arabidopsis. Interestingly, in both cases transcript oxidation was a rapid process occurring in the earliest hours of stress response and greatly preceding the over-accumulation of oxidative stress markers [49, 50]. These findings suggest that 8-OHG formation in transcripts is a primery response.

An important consequence of 8-OHG presence in mRNA is hampered translation, resulting in a decreased level of encoded proteins. The mechanism responsible for 8-OHG dependent ribosome stalling was recently described. In vitro studies carried out on reconstituted bacterial system demonstrated that 8-OHG causes a slowing down of translation process by 2–4 magnitudes. This effect was observed regardless of the position of oxidized bases in the codon, even in the wobble position. In eukaryotic extracts, translation was almost completely inhibited by the presence of 8-OHG. Formation of this modified nucleotide in transcripts leads to alterations in RNA-RNA interactions and prevents the adaptation of active conformation in the decoding center. At the same time, it has been shown that oxidized transcripts are subjected to ribosome-based quality control, and are predestined for degradation through the No-Go decay pathway (NGD) [9]. Interestingly, the translation process might be inhibited not only by the oxidation of mRNA, but also by the oxidation of rRNA. Research carried out on *E.coli* showed that hydrogen peroxide treatment led to a significant increase in 8-OHG levels in rRNA, particularly in 23S rRNA from the large ribosomal sub-unit. Some oxidation hotspots were located near the peptidyl transferase center (PCT), which is crucial for protein elongation. Subsequent experiments demonstrated that insertion of 8-oxoadenine in the A2451 position and 5-hydroxyuracyl in the U2585 position resulted in significant inhibition of translation. On the other hand, introduction of 5-hydroxycytosine in the C2063 position increased protein biosynthesis [51].

Information concerning the fate of oxidized transcripts comes mainly from studies carried out on bacterial and human models. It has been shown in HeLa cell lines that the level of oxidized RNA drops by 50% within the first hour of retraction from oxidative stress. Most probably 8-OHG rich transcripts are removed by specific ribonucleases (RNAses). Indeed some proteins engaged in the regulation of RNA stability and degradation, such as polynucleotide phosphorylase (PNPase) in *E. coli* and YB-1 protein in humans, have been found to bind to 8-OHG rich RNA with high affinity [52]. Additionally, it has been demonstrated that RNA polymerases incorporate 8-hydoxyguanosine triphosphate (8-oxoGTP) into mRNA at a significantly lower rate than its nonmodified counterpart – guanosine triphosphate (GTP). Thus, there is also a mechanism preventing the biosynthesis of 8-OHG rich transcripts through the inhibition of 8-OHG incorporation during the transcription process [53, 54].

### Nitrative modification of mRNA

Nitric oxide (NO) functions as a crucial signaling molecule in animal and plant systems, and the metabolic fate of NO gives rise to a further series of compounds, collectively known as reactive nitrogen species (RNS). One of the best recognized RNS in the cellular milieu is peroxynitrite (ONOO¯), a powerful nitrating agent formed in an extremely rapid and diffusion-controlled reaction between NO and superoxide (O_2_˙¯). It is well documented that ONOO¯ or nitrogen oxides can modify proteins, lipids and oligonucleotides, significantly affecting their biochemistry [55]. The reaction between ONOO¯ and guanine results in the production of several products, among which 8-oxoguanine and 8-nitroguanine (8-NO_2_-G) are most abundant. Thus, accumulation of 8-NO_2_-G can be considered an effective marker of nucleic acid nitration. Importantly, nitration of guanine in nucleic acids takes place at selected positions and occurs mainly at the C8 position [56, 57]. In contrast to proteins and lipids, the nitration phenomenon of nucleic acids is far from being recognized in plants.

In general, nitration of nucleotides embedded in DNA and RNA and the resulting 8-NO_2_-G formed in the presence of RNS were first indicated in hamster livers infected with liver fluke *Opisthorchis viverrini* [58] and in human gastric mucosa infected with *Helicobacter pylori* [59]. In both cases, nitration was associated with infection- or inflammation-induced carcinogenesis [60]. Although most research concerning nucleic acid nitration has been focused on DNA, it can be presumed that RNA and mRNA are more susceptible to this modification, as in the case of oxidation via ROS [61].

The search for a novel link connecting RNS and plant defense response to pathogen attacks has recently made it possible to document that the nucleic acid nitration phenomenon also occurs in plant cells. Using an immunoassay, the authors detected the presence of 8-NO_2_-G in potato leaves inoculated with *Phytophthora infestans*, a cause of late blight disease. Notably, the resistant response was accompanied by an impressive and temporally limited accumulation of nitrated RNA and mRNA pools, even in the first hour post inoculation. Unexpectedly, RNA and mRNA from the cells of the susceptible potato revealed a time-delayed and definitely lower level of 8-NO_2_-G. The temporary, vast amount of 8-NO_2_-G in the mRNA noted in the resistant response suggests that the phenomenon is rather selective and restrained by thus far unidentified control mechanisms. Based on this experiment, nitration of guanine nucleotides embedded in RNA and mRNA appears as an early switch for the redox environment facilitating a hypersensitive response (HR) during avirulent pathogen–plant interaction. Thus, targeted RNA/mRNA nitration may regulate post-transcriptional gene expression and fine-tune cell signaling that contributes to programmed cell death during HR [62]. It can be assumed that a direct consequence of nitrated bases in mRNAs is hampered translation via ribosome stalling on the transcripts, as in the case of oxidatively modified mRNA. Without a doubt, the physiological fate and identification of 8-NO_2_-G rich transcripts during plant development, responses to pathogen attack, and other stimuli await experimental verification.

## Conclusions and perspectives

Although transcript modifications are gaining increasing attention, it seems we are still in the early stages of decoding the “epitranscriptomic alphabet”. Especially in the case of plants, our knowledge seems to be fragmented. On the other hand, even the so far limited reports reveal the crucial role of transcript modifications in plants’ functioning. It has been shown that plant mRNAs are enriched by N^6^-methyladenosine (m^6^A), 5-methylcytosine (m^5^C), 8-hydroxyguanosine (8-OHG) and 8-nitroguanosine (8-NO_2_G) (summarized in Table 2). All of these modifications are implied in the regulation of developmental processes. Alterations in the formation of m^6^A result in severe defects in plants’ development. The functions of this modification are mediated by specific binding proteins, which modulate transcript stability. Similarly, decreased levels of m^5^C result in disturbances in plants’ growth and development. In turn, selective formation of 8-OHG in specific transcripts leads to hampered translation and a decrease in the level of encoded proteins. This mechanism is involved in regulating the breaking of seed dormancy.

**Table 2:** Impact of chosen mRNA modifications on plants

Modified nucleotide	Abbreviation	Plant species	Impact on plant functioning and possible involvement in stress response
N6-methyladenosine	m6A	Arabidopsis, rice	*•* m^6^A occurrence in transcripts associated with tissue specific function indicates its putative role in differentiation *• Arabidopsis thaliana* mutant with alerted methylation machinery show severe developmental defects including embryonic lethality, overgrown shoot epical meristem, changes in trichome, leaf and flower formation *•* YHT-domain proteins, known as m^6^A readers regulate lifetime of specific methylated mRNAs and are engaged in proper development of leaves and trichomes [12–35]
5-methylcytosine	m^5^C	Arabidpsis, rice, maize, barrelclover, foxtail millet	*•* Dependent on the activity of tRNA methyltransferase 4 (TRM4). The *trm4* mutants show decreased cell proliferation, hampered roots growth and increased sensitivity to oxidative stress *•* The level of m^5^C is modulated by stress factors (heat and drought) and phytohormones (auxins, abscisic acid and cytokinins) [36, 37]
8-hydoxyguanine; (8-hydroxy-2′-guanosine)	8-OHG	sunflower, wheat, soybean, Arabidopsis	*•* Selective oxidation of defined transcripts leads to hampered translation and decreased level of encoded proteins *•* Involved in breakage of seeds dormancy *•* Increase in 8-OHG levels in total RNA and/or mRNA constitutes an early response to stress factors: cadmium in soybean and nematode infection in *Arabidopsis* [47–50]
8-nitroguanine	8-NO_2_-G	Potato	*•* Overaccumulation of 8-NO_2_-G in total RNA and mRNA represents an early event preceding or coincident with the first symptoms of programmed cell death during hypersensitive response of potato leaves inoculated with *P. infestans* [62]

The functions of mRNA modifications in plants described above are firmly evidenced by experimental work. However, the results lead to many questions. The mechanism of mRNA oxidation selectivity has not yet been elucidated, neither in plants nor in animal models. Additionally, the rapid formation of 8-OHG and 8-NO_2_G in reaction to stress factors indicates that these modifications might be engaged in stress sensing. However, as of yet there are no empirical data confirming this hypothesis. In turn, m^5^C has been implicated in the regulation of plant development and stress response. Its exact role and mechanism of action remain to be discovered. Initial reports show that m^6^A binding proteins are responsible for downstream effects. Thus there is a need for identification of these proteins in various plant species and an assessment of their exact impact on transcript fate. Additionally, studies on animal and human models indicate that the role of modified ribonucleotides in cells might be even more far-reaching. For instance some reports show that in addition to modulation of mRNAs stability, m^6^A is engaged in the regulation of transcript splicing and translocation. In turn, studies on leukaemia cells indicate that complexes formed by m^5^C and specific proteins can modulate chromatin structure and its affinity to bind to biological molecules.

Moreover, taking into account that there are over 160 identified modified nucleotides, some new players might emerge in the field of plant epitranscriptomics. One potential candidate for future research is pseudouridine (Ψ). This modification is formed from uridine through the reaction of isomerization. It was discovered as early as the 1950s and was initially studied in non-coding RNA types such as rRNA and tRNA [63, 64]. However, recent reports show that Ψ is also present in mRNA. It has been detected in transcripts obtained from humans, yeast and the unicellular eukaryotic parasite *Toxoplasma gondii* [65–67]. Its formation in the mRNA of evolutionarily diverse groups of organisms indicates that it might be a universal transcript modification, possibly found in plants, as well. The impact of pseudouridylation on transcript metabolism is still not clear. However, the results obtained thus far indicate that it can affect mRNA stability (reviewed in [63, 64]). Additionally, in vitro studies have shown that incorporation of Ψ in mRNA modulates translation efficiency [68]. However, this finding has not been yet confirmed in vivo.

It is thus likely that the near future will bring new exciting discoveries in the area of plant epitranscriptomics. Further findings will greatly depend on the development and application of new sequencing methods. In the case of some modified ribonucleotides, specialized detection protocols have been already introduced. The new generation sequencing methods (NGS) used for the determination of RNA modifications can generally be divided into three classes. The first class (class I) can be applied when modified ribonucleotides cause misincorporation of nucleotides or premature termination of reverse transcription (RT). However, most of the modifications have a limited effect on RT. In this case, modified ribonucleotides can be detected using class II protocols, in which treatment with specific chemicals provokes miscorporation or RT truncation. Class II methods have been applied, for instance, for detection of Ψ and m^5^C. In the case of Ψ, treatment with soluble carbodiimide (CMCT) in alkaline conditions leads to premature RT termination. In turn, application of bisulfite leads to conversion of cytosines into uridines. Only methylated cytosines are protected from bisulfite action and can be distinguished in the process of sequencing. In class III methods, molecules that bind to modifications (usually specific antibodies) are used for precipitation of RNA fragments bearing specific modified ribonucleotide. Such a method has been applied, for example, for identification of m^6^A enriched transcripts (reviewed in [69]). Advances in sequencing will most likely be accompanied by the implementation of new bioinformatic tools, such as databases dedicated to specific modified ribonucleotides. Indeed, in addition to general databases of modified ribonucleotides, such as MODOMICS and RNAMDB [70, 71], databases directed for analysis of particular modifications, for example m^6^A, have also been recently introduced (for example [72]).

In summary, there is increasing evidence that modified ribonucleotides affect mRNA secondary structure and interaction with proteins and other RNA types, leading to changes in transcript processing, stability, localization and translation efficiency. They thus constitute a newly discovered layer of mechanisms regulating gene expression. It can be predicted that future findings in this area will have a significant impact on the drug industry, biotechnology, medicine, agriculture and nutrition [73]. It can even be postulated that we are entering a new era in biological sciences: the era of epitranscriptomics [74].

## Data Availability

All the data supporting this review is contained within the manuscript.

## Abbreviations

8-NO_2_-G: 8-niroguanosine
8-OHG: 8-hydroxyguanosine
ECT: Evolutionary Conserved C-terminal Region
m^5^C: 5-methylcytosine
m^6^A: N^6^-methyladenosine
NGS: next-generation sequencing
